# Characterization of Actinobacterial Strains as Potential Biocontrol Agents against *Macrophomina phaseolina* and *Rhizoctonia solani*, the Main Soil-Borne Pathogens of *Phaseolus vulgaris* in Cuba

**DOI:** 10.3390/plants11050645

**Published:** 2022-02-26

**Authors:** Miriam Díaz-Díaz, Alexander Bernal-Cabrera, Antonio Trapero, Ricardo Medina-Marrero, Sergio Sifontes-Rodríguez, René Dionisio Cupull-Santana, Milagro García-Bernal, Carlos Agustí-Brisach

**Affiliations:** 1 Centro de Bioactivos Químicos (CBQ), Universidad Central “Marta Abreu” de Las Villas (UCLV), Carretera Camajuaní km 5 ½, Santa Clara 54830, Villa Clara, Cuba; rpmedina@uclv.edu.cu (R.M.-M.); sifontes@uclv.edu.cu (S.S.-R.); rcupull@uclv.cu (R.D.C.-S.); mrgarcia@uclv.edu.cu (M.G.-B.); 2 Departamento de Agronomía, Unit of Excellence María de Maeztu 2020-23, Campus de Rabanales, Universidad de Córdoba, Edif. C4, 14071 Córdoba, Spain; ag1trcaa@uco.es; 3 Departamento de Agronomía, Facultad de Ciencias Agropecuarias, Universidad Central “Marta Abreu” de las Villas (UCLV), Carretera Camajuaní km 5 ½, Santa Clara 54830, Villa Clara, Cuba; alexanderbc@uclv.edu.cu; 4 Centro de Investigaciones Agropecuarias (CIAP), Facultad de Ciencias Agropecuarias, Universidad Central “Marta Abreu” de Las Villas (UCLV), Carretera Camajuaní km 5 ½, Santa Clara 54830, Villa Clara, Cuba

**Keywords:** ashy stem blight, biological control, common bean, rhizoctonia blight, *Streptomyces* spp.

## Abstract

*Macrophomina phaseolina* and *Rhizoctonia solani* are considered two major soil-borne pathogens of *Phaseolus vulgaris* in Cuba. Their management is difficult, not only due to their intrinsic biology as soil-borne pathogens, but also because the lack of active ingredients available against these pathogens. Actinobacteria, a heterogeneous bacterial group traditionally known as actinomycetes have been reported as promising biological control agents (BCAs) in crop protection. Thus, the main objective of this study was to evaluate the effectiveness of 60 actinobacterial strains as BCAs against *M. phaseolina* and *R. solani* in vitro by dual culture assays. The most effective strains were characterized according to their cellulolytic, chitinolytic and proteolytic extracellular enzymatic activity, as well as by their morphological and biochemical characters in vitro. Forty and 25 out of the 60 actinobacteria strains inhibited the mycelial growth of *M. phaseolina* and *R. solani*, respectively, and 18 of them showed a common effect against both pathogens. Significant differences were observed on their enzymatic and biochemical activity. The morphological and biochemical characters allow us to identify all our strains as species belonging to the genus *Streptomyces*. *Streptomyces* strains CBQ-EA-2 and CBQ-B-8 showed the highest effectiveness in vitro. Finally, the effect of seed treatments by both strains was also evaluated against *M. phaseolina* and *R. solani* infections in *P. vulgaris* cv. Quivicán seedlings. Treatments combining the two *Streptomyces* strains (CBQ-EA-2 + CBQ-B-8) were able to reduce significantly the disease severity for both pathogen infections in comparison with the non-treated and inoculated control. Moreover, they showed similar effect than that observed for *Trichoderma harzianum* A-34 and with Celest^®^ Top 312 FS (Syngenta^®^; Basilea, Switzerland) treatments, which were included for comparative purposes.

## 1. Introduction

The common bean (*Phaseolus vulgaris* L.) is one of the most important grain legumes in many areas of the world, providing a diet rich in protein, dietary fiber, essential micronutrients and phytochemicals for more than 500 million people [1]. The global cultivated surface of *P. vulgaris* reached 33.1 million hectares in the season 2019/2020, with an annual production of 28.9 million metric tons [2]. Common bean is the most important plant species for Cuba population within the group of edible legumes with an annual production of 169,900 tons. Together with rice (*Oryza sativa* L.), they form the basis of the daily diet in this geographic area [3].

In countries with a subtropical climate, the environmental conditions are favorable for the development and proliferation of a vast and heterogeneous soil microflora, including complexes of fungal species associated with root rot diseases such as *Alternaria alternata* (Fr.) Keissl., *Colletotrichum truncatum* (Schwein.) Andrus & W.D. Moore, *Fusarium oxysporum* Schltdl., *Macrophomina phaseolina* (Tassi) Goid., *Rhizoctonia solani* J.G. Kühn, and *Sclerotium rolfsii* Sacc. [4]. In addition, [5] pointed out that the incidence and severity of root rot diseases caused by these fungi depend on the climatic factors prevailing at each sowing time, as well as the characteristics of the microclimates existing in each region of the country where common beans are grown. Among them, *M. phaseolina* and *R. solani* are considered the most prevalent fungal pathogens associated with root rot diseases of common bean worldwide [6,7].

*Macrophomina phaseolina* (Ascomycota), causal agent of ashy stem blight, also affects roots and stems of host species via pycnidiospores and microsclerotia that persist in the soil, where the pathogen establishes the primary inoculum [8]. Typical symptoms in common bean include dark, irregular lesions on cotyledons, wilting, systemic chlorosis, premature defoliation, epinasty and early maturity or death in adult plants [6]. Late infections cause the appearance of grey areas on the stems, where microsclerotia and pycnidia of the fungus are produced. The occurrence of *M. phaseolina* in the seeds has major consequences since it causes the disqualification of legumes as propagation material [9]. For instance, six tons of common bean and three tons of broad bean to be used as planting material in the province of Villa Clara were disqualified between 2006–2007 because they were affected by *M. phaseolina* [10].

On the other hand, *R. solani* (Basidiomycota) is the causal agent of rhizoctonia blight, also commonly known as damping off [11]. This soil-borne pathogen can affect more than 500 plant species, including cultivated and wild plants, and causes damping off in stands, necrotic lesions in roots, seeds and stems, as well as foliar lesions with a worldwide distribution [7,12]. This fungus affects young seedlings much more than adult plant tissues. On the stem and hypocotyl of affected plants, reddish-brown cankers of various sizes appear, usually delimited by a dark border, which later become rough, dry up and destroy plant tissues [12]. It also attacks the roots causing foot rot of the plants [5]. The management of soil-borne pathogens including both *R. solani* and *M. phaseolina* is usually difficult, not only due to their intrinsic biology, but also because the lack of effective active ingredients. Thus, the use and extension of eco-friendly control methods such as biological control is required, not only to prevent plant diseases, but also contributing markedly to soil preservation and conservation [12].

Microorganisms belonging to genera *Bacillus* (bacteria) and *Trichoderma* (fungi) are the most commonly used biological control agents (BCAs) against soil-borne plant pathogenic fungi ([13,14]. Within this context, species belonging to *Trichoderma* fungal genus have been studied since 1930, and their use has been successfully applied directly to the soil or by seed treatments [15]. On the other hand, since the last century, bacteria belonging to the genus *Bacillus* have been used as BCAs due to their ability to colonize the rhizosphere of plants and inhibit the growth and development of plant pathogens. In addition, they are used as plant growth promoters. [14]. At the same time, the ability of these bacteria to form endospores gives them resistance to climatic changes, which is an important characteristic for inoculum production [13]. In addition to these well-known BCAs, research in the last decades highlights the benefits of the actinobacteria (*Streptomyces* spp. mainly) and their potential as BCAs (e.g., *Streptomyces griseoviridis*, *S. lydicus*) against soil-borne pathogens, such as species of *Rhizoctonia*, *Phytophthora*, *Fusarium*, and *Pythium* in legumes and other crops [16]. Actinobacteria, which have been traditionally known as actinomycetes, are a heterogeneous group of aerobic, filamentous and Gram-positive bacteria. Traditionally, the main genera isolated from soil samples are *Micromonospora*, *Nocardia*, and *Streptomyces*. The genus *Streptomyces* is represented in nature by the largest number of species among the family Actinomycetaceae [17]. This genus, as a colonizer of the rhizosphere, is able to: (i) act as BCA of plant pathogenic fungi, (ii) produce siderophores, (iii) produce plant growth promoting substances, (iv) promote nodulation, (v) produce biodegradative enzymes such as chitinases, cellulases, glucanases, peroxidases, and (vi) assist *Rhizobium* bacteria in iron assimilation, or in nitrogen fixation in legumes, which indirectly contributes to the promotion of plant growth [16]. 

As we mentioned above, ashy stem blight and rhizoctonia blight are considered the main diseases of *P. vulgaris* in Cuba since they are associated in a complex disease of this crop that causes root rot and plant death. The control management strategies already available against this complex disease are not enough for its optimum control in the frame of the sustainable agriculture. Thus, it is necessary to explore new alternatives towards biological control of these diseases. Therefore, actinobacteria could play an important role as BCAs against the main causal agents of the disease, *M. phaseolina* and *R. solani*. However, the effect of actinobacteria as BCAs against plant pathogenic fungi is still uncertain. Consequently, no biological based compounds on actinobacteria have been developed so far. Likewise, the ‘Centro de Bioactivos Químicos’ Universidad Central “Marta Abreu” de Las Villas (Cuba) has a wide collection of actinobacterial strains isolated in the central region of the country, which may be explored as a new biological alternative to be included in the integrated disease management program against soil-borne plant pathogens in the common bean crop. Therefore, the main goal of this study was to evaluate 60 actinobacterial strains for their effectiveness as BCAs against *M. phaseolina* and *R. solani* by in vitro dual-cultures assays and finally to select several actinobacterial strains with high efficiency of reduction the viability of both pathogens in vitro, and the disease progress *in planta*. We expect to select several actinobacterial strains with high efficacy on reducing the viability of *M. phaseolina* and *R. solani* in vitro, and the disease progress *in planta*.

## 2. Results

### 2.1. In Vitro Effect of Actinobacterial Strains against Macrophomina phaseolina and Rhizoctonia solani: Dual Culture Assays

**Figure 1:**
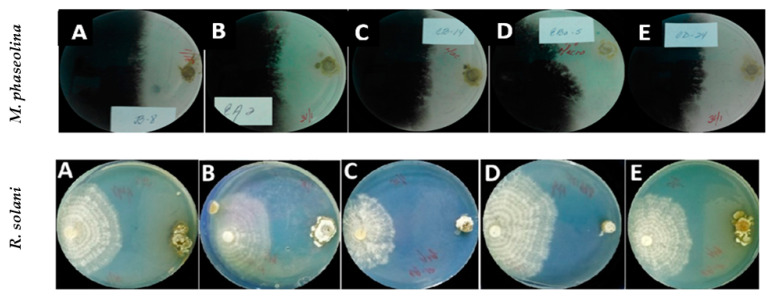
Antagonistic effect of *Streptomyces* strains against *Macrophomina phaseolina* isolate CCIBP-Mp1 (top row photos) and *Rhizoctonia solani* isolate CCIBP-Rh1 (bottom row photos) growing in dual culture on PDA at 7 days after inoculation and incubated at 28 °C in the dark. *Streptomyces* strains evaluated were: (**A**) CBQ-B-8, (**B**) CBQ-EA-2, (**C**) CBQ-CB-14, (**D**) CBQ-EBa-5, and (**E**) CBQ-CD-24 (top row photos); and (**A**) CBQ-B-8, (**B**) CBQ-CB-14, (**C**) CBQ-EA-12, (**D**) CBQ-EBa-21, and (**E**) CBQ-EA-2 (bottom row photos).

For both fungal pathogens *M. phaseolina* and *R. solani*, significant differences between actinobacterial strains were observed on their effectiveness in the Mycelial Growth Inhibition (MGI; %) (*p* < 0.001 in both cases). Regarding their effect against *M. phaseolina* isolate CCIBP-Mp1, 40 out of the 60 strains tested showed antagonistic activity against the pathogen. For this group of 40 strains, the MGI ranged from 70.4 ± 1.23 to 3.24 ± 1.01% for CBQ-EA-2 and CBQ-ESFe-11, respectively. The most effective strains against *M. phaseolina* were CBQ-EA-2, -Plat-2 and -CD-24 with mean MGI values of 70.4 ± 1.23, 66.6 ± 0.78 and 64.6 ± 1.48%, respectively. On the other hand, 25 out of the 60 actinobacterial strains tested showed antagonistic effect against *R. solani* isolate CCIBP-Rh1. For this group of 25 strains, the MGI ranged from 78.3 ± 0.37 to 5.6 ± 0.47% for CBQ-EA-12 and CBQ-C-5, respectively. The most effective strains against *R. solani* were CBQ-EA-12, -EA-2 and -CD-24 with mean MGI values of 78.3 ± 0.37, 77.4 ± 1.20 and 75.4 ± 1.22%, respectively. In addition, 19 out of the 60 actinobacteria strains evaluated showed a MGI efficacy higher than 50% for both phytopathogenic fungi, with the strains CBQ-EA-2 (MGI = 70.4 ± 1.23 and 77.4 ± 1.20% for *M. phaseolina* and *R. solani*, respectively) and CBQ-CD-24 (MGI = 64.6 ± 1.48 and 75.4 ± 1.22% for *M. phaseolina* and *R. solani*, respectively) showing the highest common effectiveness for the two pathogens. At the same time, 5% of the total actinobacteria tested did not show any effect on MGI for any of the two pathogens evaluated (Table 1; Figure 1). 

### 2.2. Qualitative Evaluation of Enzyme Activities of Actinobacterial Strains

There were significant differences between the 31 actinobacterial strains evaluated for their chitinolytic, cellulolytic or proteolytic activity (*p* < 0.0001) (Table 2). Twenty out of the 31 strains evaluated showed chitinolytic halo, which ranged from 25.3 ± 0.96 to 33.5 ± 1.91 mm for CBQ-CD-24 to CBQ-EBa-5, respectively. Concerning the cellulolytic activity, the cellulolytic halo ranged between 90.0 ± 0.41 (CBQ-B-8; -CB-14; -ECa-24; -ESFe-12; -Ni-32; -Plat-2; -Plat-3; -Plat-4; and -WP-14) and 36.3 ± 0.75 mm (CBQ-EA-3). Only three out of the 30 strains evaluated did not show cellulolytic halo (CBQ-EB-27; -EC-18; -OSS-4). Finally, 21 out of the 31 strains evaluated showed proteolytic halo, which ranged from 51.5 ± 1.50 to 27.0 ± 0.71 mm for CBQ-EA-12 to CBQ-ECa-24, respectively (Table 2).

### 2.3. Phenotypic Characterization

**Figure 2:**
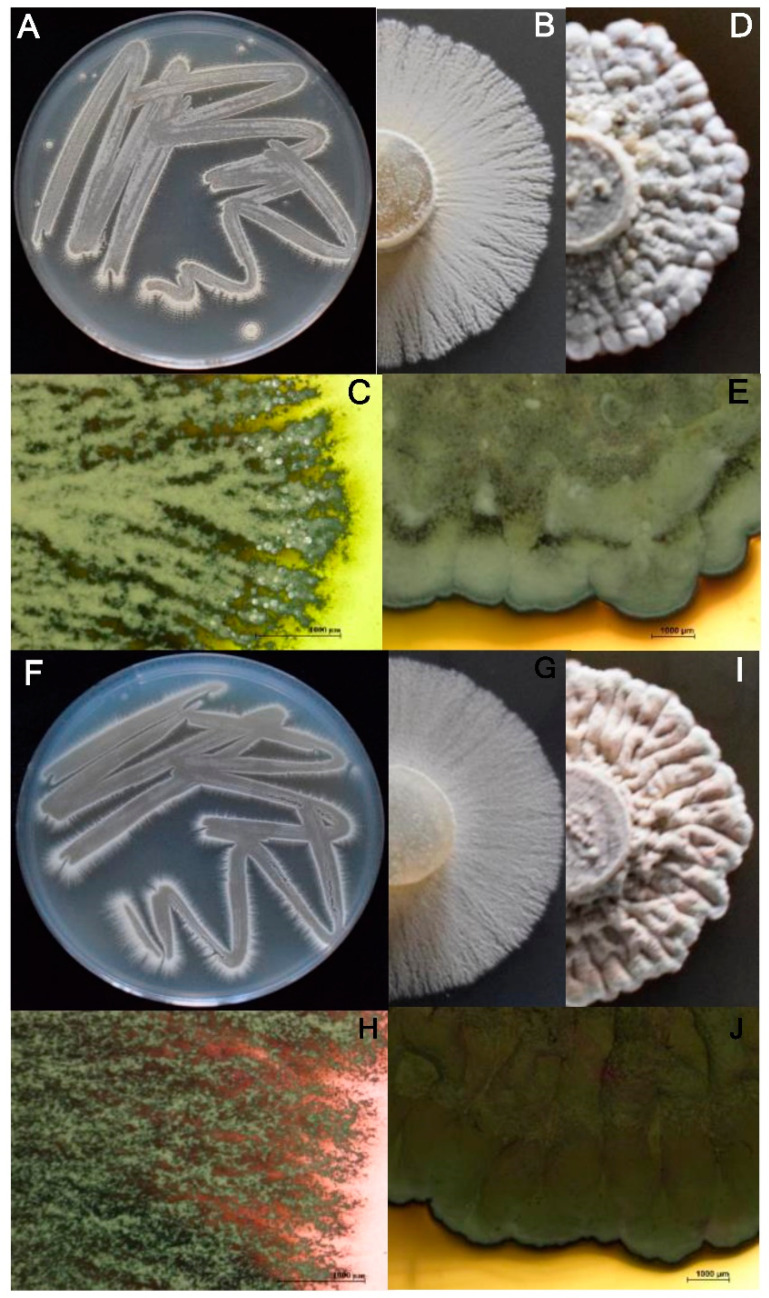
Two-weeks-old colonies of *Streptomyces* strains CBQ-B-8 (**A**–**E**), and CBQ-EA-2 (**F**–**J**) growing on ACA medium (**A**–**C**,**F**–**H**) and on PDA medium (**D**,**E**,**I**,**J**) at 28 °C in the dark.

The macroscopic features of the 11 representative actinobacterial strains selected for this experiment are show in Table 3. In general, the colonies were mostly white in color, circular in shape, convex in elevation, with an entire edge, hard consistency and variable pigment production (Figure 2). Microscopic observation of Gram-stained bacterial cells showed stable branched mycelium bearing aerial hyphae, which differentiate into short or long spore chains. Microscopic characterization using the microculture technique revealed details of aerial and vegetative mycelium, mycelial fragmentation and clustering of spores. A spiral arrangement of spores was observed on most of the microculture slides of each sample. In addition, all the strains were characterized as Gram-positive suggesting that they belong to the genus *Streptomyces*.

### 2.4. Biochemical Characterization and Assimilation of Carbon Sources

None of the eleven strains under study were positive for indole production and the Voges Proskauer test. Strains CBQ-J-4, -OSS-3, -EA-2 and -EBa-5, were positive for casein hydrolysis; and the latter two strains were also able to be positive for the methyl red test, in addition to strains CBQ-B-8, -CB-14, -EBa-21 and -Plat-2. Only the strains CBQ-EA-12 and -ESFe-4 did not hydrolyse gelatine. The strains CBQ-OSS-3 and -Plat-2 did not hydrolyse starch (Table 4). 

On the other hand, all the evaluated strains were positive for catalase citrate utilization, nitrate reduction and urea hydrolysis. Variability between strains was also observed for the assimilation and utilization of carbohydrates (Table 4).

### 2.5. Molecular Characterization

BLASTn searches on GenBank showed that the 16S rDNA sequences of the strains CBQ-EA-2 and CBQ-B-8 had 99.71 and 99.93% identity with strains of *Streptomyces* sp. HBUM206419 (MT540570) and MP47-91 (EU263063), respectively. The sequences logged in GenBank and Blast results of the two representative actinobacterial strains selected for their highest effectiveness in vitro in this study are shown in Table 5. 

### 2.6. Effect of Actinobacterial Strains against Macrophomina phaseolina and Rhizoctonia solani Infections in Planta 

Because significant differences between sterilized and non-sterilized soils, treatments, and their interaction (*p* ≤ 0.0001 in all cases) were observed on their effect on total Disease Severity (DS) (for seedlings inoculated with *M. phaseolina*) and on DSstem and DSroot (for seedlings inoculated with *R. solani*), individual ANOVA per each type of soil was conducted to evaluate the effect of treatment on DS of each tissue. 

#### 2.6.1. Effect of Treatments against *Macrophomina phaseolina in Planta*

**Figure 3:**
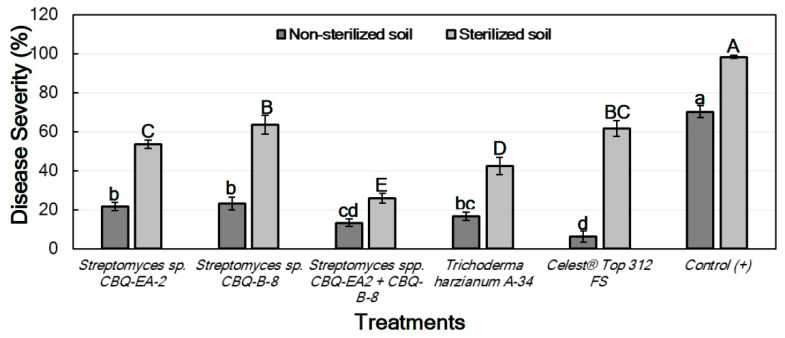
Disease severity (%) in *Phaseolus vulgaris* cv. Quivicán seedlings treated with biological or chemical compounds and inoculated with *Macrophomina phaseolina* isolate CCIBP-Mp1 at 35 days growing on non-sterilized or sterilized soil. Each column represents the mean of 40 seedlings per soil and treatment combination. Columns with a common uppercase or lowercase letter do not differ significantly according to Fisher’s protected LSD test (*p* = 0.05) for treatments on non-sterilized or sterilized soil, respectively. Vertical bars are the standard errors of the means.

For the treatments conducted with seedlings grown in non-sterilized soil, significant differences between treatments were observed for their effect on DS (*p* ≤ 0.0001). DS ranged from 21.7 ± 2.1 to 6.4 ± 2.8% for seedlings treated with *Streptomyces* sp. CBQ-EA-2 and Celest^®^ Top 312 FS, respectively, with all treatments showing a significant effect on the disease progress in comparison with the non-treated and inoculated seedlings (positive control; DS = 70.3 ± 3.1%) (Figure 3).

Concerning the treatments conducted with seedlings grown in sterilized soil, significant differences between treatments were also observed for their effect on DS (*p* ≤ 0.0001). In this case, all treatments also resulted in significant effectiveness compared to the positive control (DS = 98.3 ± 0.7%). DS among treated seedlings ranged from 63.6 ± 4.9 to 25.8 ± 2.5 for treatments with *Streptomyces* sp. CBQ-B-8 and *Streptomyces* sp. CBQ-EA-2+ CBQ-B-8, respectively (Figure 3).

**Figure 4:**
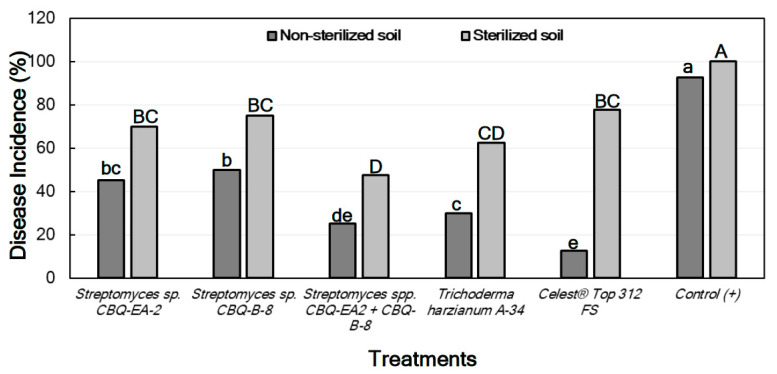
Disease incidence (DI, %) in *Phaseolus vulgaris* cv. Quivicán seedlings treated with biological or chemical compounds and inoculated with *Macrophomina phaseolina* isolate CCIBP-Mp1 at 35 days growing on non-sterilized or sterilized soil. Each column represents the mean of 40 seedlings per soil and treatment combination. Columns with a common uppercase or lowercase letter do not differ significantly according to Dunn’s multiple comparisons for proportions test (*p* = 0.05) for treatments on non-sterilized or sterilized soil, respectively.

The Disease Incidence (DI) was markedly lower in treated seedlings grown in non-sterile soil than those grown in sterile soil. In both cases, not only there were significant differences in DI between treatments, but also significant differences were observed between all the treatments and the positive control (*p* ≤ 0.0001 in all cases), the latter always showing the highest DI values. In all cases, the treatments with *Streptomyces* sp. CBQ-EA-2 + CBQ-B-8, *T. harzianum* A-34, or Celest^®^ Top 312 FS showed the lowest DI values (Figure 4). No seedling mortality was not observed in any case, except for the positive control grown in sterile soil which presented 100% mortality.

#### 2.6.2. Effect of Treatments against *Rhizoctonia solani in Planta*

**Figure 5:**
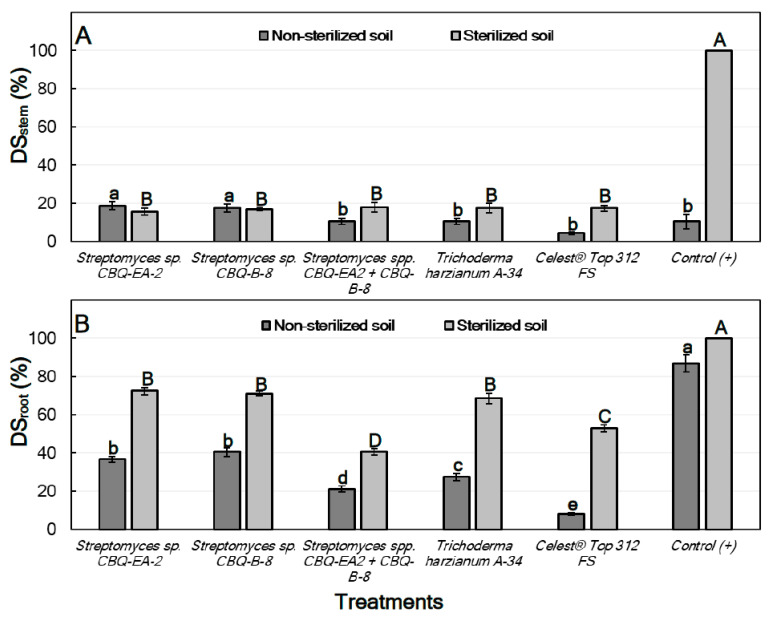
Disease severity (%) in stem (**A**; DSstem) and roots (**B**; DSroot) of *Phaseolus vulgaris* of cv. Quivicán seedlings treated with biological or chemical compounds and inoculated with *Rhizoctonia solani* isolate CCIBP-Rh1 at 28 days growing on non-sterilized or sterilized soil. Each column represents the mean of 40 seedlings per soil and treatment combination. Columns with a common uppercase or lowercase letter do not differ significantly according to Fisher’s protected LSD test (*p* = 0.05) for treatments on non-sterilized or sterilized soil, respectively. Vertical bars are the standard errors of the means.

For treatments conducted with seedlings grown in non-sterilized soil, significant differences between treatments were observed for their effect on both DSstem (*p* = 0.0015) and DSroot (*p* ≤ 0.0001). In all cases, DSstem was lower than DSroot, ranging from 18.7 ± 2.21 to 4.4 ± 0.77% for seedlings treated with *Streptomyces* sp. CBQ-EA-2 and Celest^®^ Top 312 FS, respectively. But no important differences were observed for their effect on DSstem compared to the positive control (DSstem = 10.6 ± 3.78%). However, all the treatments showed significantly higher effectiveness on DSroot compared to the positive control (DSroot = 86.8 ± 4.38%). Treatments with *Streptomyces* sp. CBQ-EA-2 (DSroot = 36.9 ± 1.53%) or CBQ-B-8 (DSroot = 40.6 ± 2.21%) were the least effective, while the treatment that combined the two strains was highly effective (DSroot = 21.2 ± 1.53%) showing results similar to those observed for *T. harzianum* A-34 (DSroot = 27.5 ± 2.07%) (Figure 5).

Regarding the treatments conducted with seedlings grown on sterilized soil, significant differences were also observed between treatments for their effect on both DSstem (*p* ≤ 0.0001) and DSroot (*p* ≤ 0.0001). In this case, all the treatments were highly effective compared to the positive control (DSstem = 100%), but no significant differences in effectiveness between treatments were observed. DSstem ranged from 18.1 ± 2.50 to 15.6 ± 1.71% for treatments with *Streptomyces* sp. CBQ-EA-2+ CBQ-B-8, and with *Streptomyces* sp. CBQ-EA-2, respectively. On the other hand, all the treatments also showed significantly lower DSroot values compared to the positive control (DSroot = 100%), but significant differences were also observed between treatments for their effect against the disease. The most effective treatment was *Streptomyces* sp. CBQ-EA-2+ CBQ-B-8 (DSroot = 40.6 ± 2.21%), and the least effective were *Streptomyces* sp. CBQ-EA-2 (DSroot = 72.5 ± 1.82%), *Streptomyces* sp. CBQ-B-8 (DSroot = 71.3 ± 1.17%) and *T. harzianum* A-34 (DSroot = 68.7 ± 2.62%) (Figure 5).

**Figure 6:**
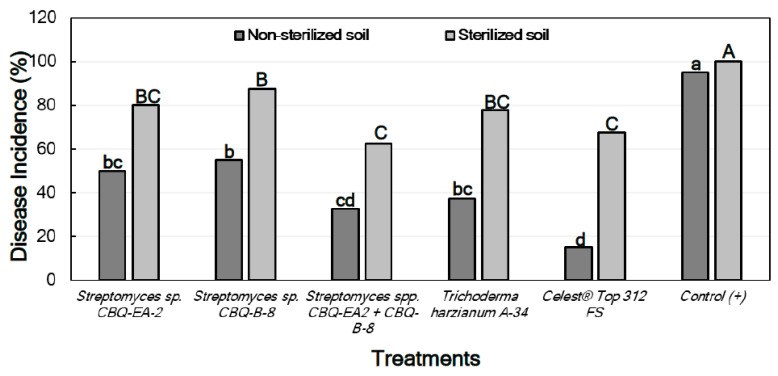
Disease incidence (DI, %) in *Phaseolus vulgaris* cv. Quivicán seedlings treated with biological or chemical compounds and inoculated with *Rhizoctonia solani* isolate CCIBP-Rh1 at 28 days growing on non-sterilized or sterilized soil. Each column represents the mean of 40 seedlings per soil and treatment combination; and columns with a common uppercase or lowercase letter do not differ significantly according to Zar’s multiple comparisons for proportions test (*p* = 0.05) for treatments on non-sterilized or sterilized soil, respectively.

A pattern similar to that observed for seedlings inoculated with *M. phaseolina* was found for the effect of the treatments on the DI of seedlings inoculated with *R. solani*, with significant differences in DI being between all treatments and the positive control (*p* ≤ 0.0001 in all cases). In all cases, the treatment with *Streptomyces* sp. CBQ-EA-2 + CBQ-B-8 showed significantly less DI than the treatments with *Streptomyces* sp. CBQ-EA-2 or *Streptomyces* sp. CBQ-B-8, and also showed DI values similar to those observed for *T. harzianum* A-34 and Celest^®^ Top 312 FS (Figure 6). 

Finally, for both lots of plants growing in non-sterilized and sterilized soil, the linear correlation analysis showed that there was not significant linear correlation between DSstem and DSroot (non-sterilized soil: *r* = 0.2869; *p* = 0.5815; sterilized soil: *r* = 0.7739; *p* = 0.0709), and DSstem and DI (non-sterilized soil: *r* = 0.3826; *p* = 0.4541; sterilized soil: *r* = 0.7409; *p* = 0.0920). Nevertheless, a significant positive linear correlation was observed between DSroot and DI in both non-sterilized soil (*r* = 0.9944; *p* = 0.0001) and sterilized soil (*r* = 0.9710; *p* = 0.0013).

## 3. Discussion

Actinobacteria (*Streptomyces* spp. mainly) have been reported as potential BCAs against soil-borne pathogens of legumes during the last decade [16]. However, the use of actinobacteria as BCAs in the frame of the integrated management of the major diseases of common bean caused by soil-borne pathogens in Cuba has not been explored yet. Therefore, this study aimed to characterize a collection of 60 actinobacterial strains from Cuba based on their in vitro effectiveness against the two main soil-borne pathogens of common bean in Cuba, as well as on their phenotypic and biochemical characteristics. 

All the selected actinobacteria formed a smooth surface colony in CAS, becoming white to beige, hard and compact with age, varying in pigmentation, powdery or velvety appearance as a result of the formation of short and long chains of spores, with typical smell of wet soil (Figure 2). In the totality of the microcultures a spiral arrangement of the spores was observed. Similar results were obtained by Ayuningrum and Jati [20], whom reported that isolates of actinobacteria forming powdery colonies with well-developed aerial hyphae divided into spore chains were termed *Streptomyces*-like actinomycete bacteria. This fact together with the concordance of the morphological characters of our strains with those described by Bergey [19] for the *Streptomyces* genus, indicate that all of our actinobacteria strains belong to this genus. In addition, our *Streptomyces* strains showed high levels of cellulolytic and proteolytic activity. Our results are also in concordance with those previously obtained by several authors, who reported the ability of *Streptomyces* strains to produce high levels cellulase and protease [21]. For instance, 62% of our *Streptomyces* strains revealed a high cellulolytic capacity with a halo between 80 to 90 mm in diameter, and 90% of them developed a halo with considerable extension around the colony, which denotes an important cellulolytic hydrolysis. Similar results were recently obtained by Rani et al. [22], who reported that the 67.5 and 60.0% of the *Streptomyces* isolates of their collection showed cellulolytic and proteolytic activity, respectively. Furthermore, the 66.7% of our *Streptomyces* strains showed chitinolytic capacity, highlighting the CBQ-EBa-5 strain, with a 35.5 mm clearance halo surrounding the colony. These results are also in agreement with those obtained by Liu et al. [23], who showed that *S. hydrogenans* (SSD60) and *S. spororaveus* (SDL15) had strong chitinolytic activity, and the 24% of the *Streptomyces* strains of their collection (*n* = 94) developed a clear halo surrounding the colony when evaluating their chitinolytic activity. Altogether, it not only confirms that our strains are well identified as *Streptomyces*, but also suggests that the actinobacteria form one of the most important microbial communities in soil rehabilitation and conservation, as they are largely responsible for their ability to produce extracellular cellulolytic, chitinolytic and proteolytic enzymes. 

Actinobacteria represent a source of biologically active secondary metabolites, including enzymes [24]. In this study, we achieved specific qualitative metabolic characterization such as enzymatic, biochemical, morphological and antagonistic of at least 11 strains, which is the main criterion for determining their environmental role and their action in biogeochemical cycles. The challenge of our future research has its origins in this study, so evaluating the in vitro antagonistic activity of our strains showed that many of them disseminate secondary metabolites in the same culture medium in which they inhibit the growth of *M. phaseolina* and *R. solani*. After having evaluated the enzymatic activities, we could infer that the production of chitinases has a positive effect in this regard, since chitin is one of the major components of the fungal cell wall. In addition, actinobacteria combine with other soil microorganisms in their natural environment to decompose resistant plant debris, such as cellulose, as well as animal debris to maintain the biotic balance of the soil by cooperating with the nutrient cycle [25]. Although we were able to identify well all of our actinobacterial strains as *Streptomyces* spp. based on their phenotypic and biochemical characters, the identity of the two representative strains that showed that highest effectiveness on MGI in vitro in this study (CBQ-EA-2, and -B-8) was confirmed by sequencing the 16S rRNA gene using the universal primers 27f and 1492r for eubacteria. The consensus sequences obtained were blasted in GenBank and they match with more than 99% of maximum identity with reference sequences from *Streptomyces* spp. According to Law et al. [26], the 16S rRNA gene has been extensively studied with proven sensitivity for taxonomic and phylogenetic identification of most bacteria including actinobacteria such as *Streptomyces* spp. 

Regarding the in vitro efficacy of our 60 *Streptomyces* potential strains against *M. phaseolina* and *R. solani*, it varied depending on the soil-borne pathogen tested. It is worth mentioning that 40 and 25 out of the 60 actinobacterial strains inhibited the mycelial growth of *M. phaseolina* and *R. solani*, respectively. Among the most effective strains, 18 of them showed a common effect against both pathogens, with the CQB-EA2, and -CD-24 being among the strains that showed greater efficacy in inhibiting mycelial growth of the two pathogens. Our results are similar than those described by Dalal et al. [27], who evaluated in vitro the antagonistic activity of 15 strains of actinobacteria against various soil-borne soybean pathogens. These authors reported that the 15 strains showed some effectiveness in inhibiting the mycelial growth of *R. solani*, and six of the 15 strains were also able to inhibit mycelial growth of *M. phaseolina* [25]. Similarly, Singh et al. [28] evaluated the antifungal activity of 80 strains of actinobacteria against *C. truncatum*, *F. oxysporum*, *M. phaseolina*, and *S. rolfsii*, highlighting the greater efficacy of *Streptomyces* sp. strain ACITM-1 on inhibition of mycelial growth of all pathogens. In addition to these, several *Streptomyces* sp. strains has also been reported for their high efficacy in inhibiting the mycelial growth of soil-borne pathogenic fungi, such as *R. solani* [29], *R. bataticola* [30], *M. phaseolina* [21], *F. oxysporum*, *Alternaria* sp., and *Magnaporthe oryzae* [21]. 

Finally, seed treatments with *Streptomyces* sp. CBQ-EA-2 and -B-8 were evaluated separately and in combination against infections by *M. phaseolina* and *R. solani* in inoculated seedlings of common bean under semi-controlled conditions. In general, the treatments conducted using a mix of the two *Streptomyces* sp. strains (CBQ-EA-2 + -B-8) showed a significant greater effectiveness against both pathogens compared to treatments performed with the two strains alone. In addition, the effectiveness of the two combined *Streptomyces* strains in controlling the disease was similar to that observed for the other comparative treatments such as *T. harzianum* A-34 or the chemical (Celest^®^ Top 312 FS). Interestingly, the DS was higher in seedlings grown in sterilized soils than in those grown in non-sterilized soils, also varying the effectiveness of the different treatments with the soil used. It suggests that the microbiota of the soil is in active and positive interaction with the plant and the pathogen, making difficult the pathogen infection and development. Further research to evaluate the effect of the microbiota of the soils used in this study on the biology of both *M. phaseolina* and *R. solani* should be conducted to determine the potential plant-soil-pathogen interactions.

Our results are in concordance with those reported by Yadav et al. [31], who showed that *Streptomyces* sp. S160 reduced the incidence of charcoal rot caused by *M. phaseolina* under greenhouse conditions in chickpea by 33.3% relative to the control. Similarly, Alekhya et al. [32] found that *Streptomyces* sp. (BCA-546 and CAI-8) significantly reduced charcoal rot in sorghum caused by *M. phaseolina* under semi-controlled conditions. On the other hand, our results are also in correspondence with those reported by Korayem et al. [33] who evaluated the biological activity of *S. parvulus* strain 10d against *R. solani* on green beans in a semi-controlled trial with sterilized and non-sterilized soil. These authors showed that seedlings plants treated with a spore suspension of *S. parvulus* strain 10d showed the highest survival rate (88%) and the lowest DSroot (28%) in the whole of the experiment, showing much better results than those observed for seedlings treated with specific chemicals such as Rhizolex^®^ [31]. Similarly, Fatmawati et al. [29] evaluated 10 strains of actinobacteria against *R. solani* on soybean seeds under controlled conditions, with *Streptomyces* spp. strain ASR53 showing the best results in suppressing damping-off disease by 68% and 91% in sterile soil and non-sterile soil, respectively.

This study represents the first report evaluating the effect actinobacteria against the main soil-borne pathogenic fungi of common bean in Cuba. It also shows that *Streptomyces* spp. should be considered as possible biocontrol alternatives against soil-borne pathogens, not only for their effectiveness in disease control, but also for their role in soil preservation which is highly recommended in the frame of sustainable agriculture. Due to the conclusions of this study are based on experiments under controlled conditions, the most effective *Streptomyces* strains of this study may be evaluated against the disease under natural field conditions in the future. Altogether will help us to develop potential BCAs for the control of *M. phaseolina* and *R. solani* associated with stem and root-rot diseases of common bean in Cuba.

## 4. Materials and Methods

### 4.1. Actinobacterial Strains and Growth Conditions

A total of 60 actinobacterial strains isolated from different substrates or geographical areas of west-central Cuba were included in this study. They were recovered from rhizosphere (21), stem (15) or root (9) samples from a wide diversity of hosts, among other sources (Table 6), and stored in the laboratory at 4 °C for no more than 72 h until processing. For isolation of actinobacteria from rhizosphere samples, 1 g of each sample was suspended in 9 mL of sterile distilled water (SDW) by vortexing and incubated in water bath at 55 °C for 6 min. Subsequently, serial dilutions (up to 10–5) were performed. The same procedure was carried out with stem or root samples, but they were previously macerated in a mortar with sterile sand. In all cases, 100 µL aliquots of each dilution were spread in 9.0 cm diameter Petri dishes containing casein-starch agar (CSA) supplemented with filtered cycloheximide (100 µg/mL) and nalidixic acid (30 µg/mL) [34]. The inoculated Petri dishes were incubated at 28 °C for 28 days in darkness. Based on macroscopic characters i.e., texture, appearance, surface with or without aerial mycelium, colonies of actinobacteria were selected, transferred to CSA, and incubated as described before. Subsequently, spore suspensions were obtained from the pure cultures of each selected strain, and they were kept in 2 mL translucent screw-capped microtubes (Zhejiang Runlab Technology Co., Taizhou, China) at −20 °C in 20% glycerol for further studies [35]. The collection belongs to the Microbiology Laboratory of the CBQ of the Universidad Central “Marta Abreu” de Las Villas (Cuba).

### 4.2. In Vitro Effect of Actinobacterial Strains against Macrophomina phaseolina and Rhizoctonia solani: Dual Culture Assays

All the 60 actinobacterial strains (Table 6) were evaluated for their effectiveness inhibiting mycelial growth of *M. phaseolina* isolate CCIBP-Mp1 and *R. solani* isolate CCIBP-Rh1 by means in vitro dual culture assays. The two pathogenic fungi were obtained from the collection of plant pathogenic fungi of the Instituto de Biotecnología de las Plantas (IBP) of the Universidad Central Marta Abreu de Las Villas (Cuba), where are maintained growing on PDA at 5 °C in darkness. These isolates were selected due to their high aggressiveness previously tested in the common bean crop [36].

Prior to conduct the dual culture assay, the 60 actinobacterial strains were grown on CSA (pH = 7) at 30 °C for seven days in darkness. The inoculum of *M. phaseolina* and *R. solani* was prepared by seeding suspensions of mycelial fragments of each isolate on Potato Dextrose Agar (PDA; BioCen, Bejucal, Mayabeque, Cuba) at 28 °C for three days in darkness. In vitro dual culture assays were conducted in 9.0 cm in diameter Petri dishes with PDA [37]. To this end, a 7.0 mm in diameter mycelial plug of the pathogen was placed at one end of the plate, and another 7.0 mm in diameter mycelial plug of the actinobacterial strain was plated at 50.0 mm apart at the opposite end. Additionally, 7.0 mm in diameter mycelial plugs of *M. phaseolina* or *R. solani* isolates were seeded in the center of PDA plates without actinobacteria as a positive growth control. All Petri dishes were incubated at 28 °C in total darkness, and the radial mycelial growth of the two plant pathogens was assessed every 24 h, until seven days of incubation [38,39]. There were three replicated Petri dishes per actinobacterial strain (*n* = 60) and plant pathogen (*n* = 2) or control (*n* = 2) combination in a completely randomized design [(60 actinobacterial strains × 2 fungal pathogens × 3 Petri dishes) + (2 control × 3 Petri dishes) = 366 Petri dishes in total]. The experiment was performed three times under similar conditions.

For each fungal pathogen, the percentage of the inhibition of mycelial growth was calculated using the following formula:

Mycelial growth inhibition (MGI) (%) = [(RGR-rgr)/RGR] × 100

where ‘rgr’ is the radial growth of *M. phaseolina* or *R. solani* in dual culture with each actinobacterial strain, and ‘RGR’ is the radial growth rate of the control treatment (fungal pathogen isolates growing on PDA without actinobacterial strains).

### 4.3. Qualitative Evaluation of Enzyme Activities of Actinobacterial Strains

Of the 60 strains analyzed in vitro (4.2), the 31 most effective were selected to determine their chitinolytic, cellulolytic, proteolytic activity (Table 6). For chitinolytic activity, all the strains were grown on Colloidal Chitin Agar culture medium (pH = 7) at 28 °C for seven days in darkness [40]. For cellulolytic activity, the strains were grown on ISP2 (International Streptomyces Project) [41] with cellulose (1%, *w*/*v*) (pH = 7.2) also at 28 °C for seven days in darkness; then a congo red solution (1%) was added as developer for 15 min; and finally, the congo red solution was removed and NaCl solution (1 M) was added for 15 min [42]. For proteolytic activity, the strains were grown on ISP2 with 1% skimmed milk at 30 °C for seven days in darkness [43]. For each parameter evaluated, there were three replicated Petri dishes per strain in a completely randomized design (93 Petri dishes in total), and the experiment was performed three times under similar conditions. 

In all cases, after seven days of incubation, the halo surrounding the colonies of the actinobacterial strains was measured (mm) from the center of the inoculated mycelial disc.

### 4.4. Phenotypic Characterization

Taking into account the macroscopic appearance of the 60 actinobacterial strains evaluated for their effectiveness on MGI of the two pathogens in this study, a total of 11 strains (Table 6) were selected as representative of the main groups with slightly differences on the colony morphology to complete their macro- and microscopic morphological characterization. These strains were grown on CSA as described above, and then, macroscopic colony characters such as presence and color of aerial mycelium, as well as substrate color, shape, elevation, edges and consistency of colonies were recorded [18,19]. Subsequently, microscopic observations were conducted under optical microscope (LABOMED^®^, Fremont, CA, USA). Bacterial cell observations were carried out on fresh and stained preparations (simple and Gram staining) to define the shape, clustering and response to Gram stain [19]. Additional microscopic features such as aerial and vegetative mycelium, mycelial fragmentation, or clumping of spores were recorded by microcultures with lactophenol blue as a contrast stain [44], and they were compared with those described in Bergey’s Manual of Bacteriological Determination [45]. There were three replicated Petri dishes per strain in a completely randomized design (33 Petri dishes in total), and the experiment was performed three times under similar conditions.

### 4.5. Biochemical Characterization and Assimilation of Carbon Sources

The biochemical characterization using traditional techniques of the same 11 actinobacterial analyzed in the Section 4.4 (Table 6) was evaluated by applying the following tests: catalase, acid production by using different carbohydrate sources (e.g., glucose, mannitol, dextrose, fructose, maltose, raffinose, sucrose and xylose), casein hydrolysis, citrate utilization, indole test, and gelatin hydrolysis [46]. The ability to produce hydrolytic enzymes for the utilization of polysaccharides such as starch was also determined. The hydrolysis of urea to reveal the activity of the enzyme urease [47], methyl red (MR) and Voges Proskauer (VP) tests were carried out according to the ISP [18]. There were three replicated Petri dishes per strain in a completely randomized design (33 Petri dishes in total), and the experiment was performed three times under similar conditions.

### 4.6. Molecular Characterization

The actinobacterial strains CBQ-EA-2 and CBQ-B-8 were grown in tryptone-soya broth (BioCen) at 30 °C for three days, and centrifuged at 16,000 rpm. DNA was extracted from the resulting pellet using the PureLink™ Genomic DNA Mini Kit reagent (Invitrogen, Waltham, MA, USA), following the manufacturer’s instructions. The universal primers 27f and 1492r [48] for eubacteria were used to amplify the 16S rRNA gene via Polymerase Chain Reaction (PCR). Each reaction mixture contained each primer at 20 µM, dNTPs at 10 µM, 5 µL of 10X MgSO_4_ and buffer, dimethyl sulfoxide (5%), 1 µg of genomic DNA and 1 unit of taq DNA polymerase, for a final volume of 50 µL. PCR steps included an initial denaturation at 94 °C for 3 min, followed by 30 cycles at 94 °C for 30 s, 47 °C for 33 s and 72 °C for 90 s and a final extension step at 72 °C for 7 min. PCR products were run through 1% agarose gel electrophoresis stained with RedSafe™ dye (iNtRONBiotechnology), followed by purification using the PureLink™ kit (Invitrogen, Waltham, MA, USA) and determination of amplicon quality by spectrophotometry (NanoDrop 2000, ThermoScientific; Waltham, MA, USA). Sequencing was carried out on the ABI310 Prism automated sequencer (Applied Biosystems; Waltham, MA, USA), and the resulting sequences were compared with those in the GenBank database using the BLAST (Basic Local Alignment Search Tool) algorithm to identify closely related sequences [49,50]. The consensus sequences were uploaded to GenBank data base (Table 6).

### 4.7. Effect of Actinobacterial Strains against Macrophomina phaseolina and Rhizoctonia solani Infections in Planta 

#### 4.7.1. Plant Material

Seedlings of the common bean (*P. vulgaris* L.) of cv. Quivicán (white testa) were used in this study. The seeds used are registered in the official list of commercial cultivars [51] from the ‘UEB Semillas Villa Clara’. Prior to conduct the experiments, the viability of seeds was tested estimating the percentage of germination (%) using a humid chamber at 100% of relative humidity (RH). The seeds were previously disinfected in a serial wash by dipping them first in a 70% ethanol solution for 5 min, then in a 1.5% sodium hypochlorite solution for 15 min, and finally, three times in distilled water for 20 min.

#### 4.7.2. Biological Control Agents and Inoculum Preparation

The actinobacterial strains CBQ-EA-2 and CBQ-B-8 were selected to conduct the experiments *in planta* because they were considered as representative of the strains showing high (CBQ-EA-2; MGI = 70.4 and 77.4% for *M. phaseolina* and *R. solani*, respectively) and moderate (CBQ-B-8; MGI = 63.1 and 69.0% for *M. phaseolina* and *R. solani*, respectively) effectiveness to both pathogens in the dual culture assays. In addition, their morphological, biochemical, and extracellular enzymatic characteristics together with their molecular characterization were also taken into account to ensure that they belong to *Streptomyces* genus together. To prepare the inoculum of the two strains for seed treatments (see below), 20 µL of the original spore suspension preserved at −20 °C in 20% glycerol were firstly added in a 5 mL sterile plastic tubes with tryptone soy broth (BioCen) and incubated at 28 °C for 48 h [52]. Then, they were transferred to 250 mL Erlenmeyer flasks with 100 mL of tryptone soy broth and shaken in a Gerhardt orbital shaker at 28 °C at a speed of 120 G for 3 days. Finally, the inoculum of each actinobacterial strain was adjusted at 1 × 10^8^ spores mL^−1^ using a hemocytometer.

Additionally, *Trichoderma harzianum* strain A-34 belonging to the Plant Health Research Institute (INISAV, La Habana, Cuba) was also included in this experiment as a BCA for comparative purposes. The selected strain is the active ingredient of a bioproduct for the control of phytopathogenic soil fungi, foliar diseases and nematodes commonly used in Cuba [53]. To prepare the inoculum of *T. harzianum* A-34 for seed treatments (see below), sterile 250 mL Erlenmeyer flasks with 100 mL of Potato Dextrose Broth (PDB; BioCen) were inoculated by adding five 10-mm in diameter mycelial plugs of *T. harzianum* A-34 obtained from the active margin of colonies previously grown on PDA at 28 °C in darkness for 72 h. Then, the inoculated Erlenmeyer flask were shaken as described above, and the inoculum was adjusted at 1 × 10^8^ spores mL^−1^. 

#### 4.7.3. Soil Inoculation with *Macrophomina phaseolina* and *Rhizoctonia solani*

The effectiveness of the selected BCAs was evaluated in planta against *M. phaseolina* isolate CCIBP-Mp 2, and *R. solani* isolate CCIBP-Rh1. To prepare the inoculum of both isolates, 1-L Erlenmeyer flasks were filled with 200 g of an artificial substrate (risk husk, part rice grain and distilled water; 3:1:0.5, weight:weight:volume) and sterilized at 120 °C for 1 h. Subsequently, the flasks were seeded with five 1.0-cm in diameter of mycelial plugs of *M. phaseolina* isolate CCIBP-Mp 2 or *R. solani* CCIBP-Rh1 anastomosis groups (AG-4_HGI), taken from the edge of the active growing colonies previously grown on PDA as described before. The inoculated flasks were incubated at 28 °C in darkness for 10 days, and they were manually shaken each 2 days to favor the homogeneous colonization of the substrate [54]. In this study, a medium washed fluffy brown soil [55] non-sterilized and sterilized (120 °C for 20 min in cycles of three consecutive days, and subsequent sterility testing) was used in this study. In all cases, and for each pathogen, the inoculation was carried out at 2% by homogenizing the colonized substrate with the soil [56]. 

Subsequently, plastic pots were filled with 1.5 Kg of this mix. After 48 h of mix preparation (soil + colonized substrate), four common bean seeds previously treated were sown per plastic pot, and soil moisture was kept at 80% of the field capacity (FC).

#### 4.7.4. Seed Treatments, Growth Conditions and Experimental Design

Seed treatments were conducted by dipping the seeds for 30 min in the following suspensions: (i) actinobacterial strain CBQ-EA-2 at 1 × 10^8^ spores mL^−1^; (ii) actinobacterial strain CBQ-B-8 at 1 × 10^8^ spores mL^−1^; (iii) a mix of the actinobacterial strains CBQ-EA-2 and CBQ-B-8 at 1 × 10^8^ spores mL^−1^ global concentration; (iv) *T. harzianum* strain A-34 at 1 × 10^8^ spores mL^−1^; and (v) Celest^®^ Top 312 FS (Syngenta^®^; Basilea, Switzerland) prepared in a water suspension of 192 mL of active ingredient per kg of seeds. The latter chemical compound was included for comparison purposes. Additionally, seeds dipped for 30 min in SDW were also included as non-treated control seeds, and lots of non-treated seeds were sowed in plastic pots with inoculated soil (treatment (vi): positive control) as well as in plastic pots with non-inoculated soil (treatment (vii): negative control).

After more than 50% of the seeds emerged, seedlings were treated every three days by wetting the substrate with 1 mL of the respective biological treatment (actinobacterial or *T. harzianum*) adjusted to 1 × 10^8^ spores mL^−1^ until the end of the experiment [28 days after sowing (das)]. Both positive and negative controls and the chemical treatment were wetted every three days with 1 mL of SDW.

For each pathogen, a split-plot design was used with soil (*n* = 2; sterilized and non-sterilized) as the main plot factor and treatments (*n* = 7) as sub-plot factor; with ten pots (replicates) per treatment, and 4 seeds per replicate (*n* = 40). They were maintained in a CBQ greenhouse at 28 °C, 70% RH and 1100 μmol m^−2^ s^−1^ light intensity.

#### 4.7.5. Disease Severity Assessment 

For treated seedlings inoculated with *M. phaseolina*, disease severity (DS) was assessed at 35 days after inoculation using the following DS rating scale: 1 = no visible disease symptoms; 3 = wilt restricted to cotyledons, lower stem tissues with small necrotic lesions; 5 = 10% of hypocotyl and lower stem tissues showing lesions, fungal fruiting structures starting the development in the affected tissues, 7 = 25% of hypocotyl and lower stem tissues showing lesions, with development of fungal fruiting structures in the affected tissues; and 9 = ≥50% of hypocotyl and lower stem tissues with lesions, with abundant development of fungal fruiting structures [57]. Subsequently, a DS index was estimated using the following formula:

$$DS(\%)=[\overset{9}{\underset{i=1}{\sum }}n_{i}\left(st_{i}\right)/(N\times K)]\times 100$$

in which *n_i_* = number of seedlings in the DS development stage *i*, *st_i_* = value of the DS stage (1–9), *N* = total number of plants assessed, and *K* = largest scale level (9) [58]. 

Regarding treated seedlings inoculated with *R. solani*, DS was evaluated separately on stem and roots tissues at 28 days after inoculation by using the following DS rating scales: (i) DSstem: 1 = absence of lesions on hypocotyl, 2 = superficial lesions (yellow-brown discoloration) on hypocotyl, 3 = deep tissue lesions, and 4 = seedlings dead or wilted [59]; (ii) DSroot: 0 = healthy seedlings, 1 = yellowish-brown discoloration near hypocotyl, 2 = yellowish-brown discoloration plus lesions or brown spots near hypocotyl, 3 = entirely brown surface or lesions covering more than 75% of root surface, and 4 = pre-emergence damping off, seedlings dead or wilted [60]. Subsequently, a DS index was estimated for each tissue using the following formulas:

$$\mathrm{DSstem}(\%)=[\overset{4}{\underset{i=1}{\sum }}n_{i}\left(st_{i}\right)/(N\times Kstem)]\times 100$$

$$DSroot(\%)=[\overset{5}{\underset{i=1}{\sum }}n_{i}\left(st_{i}\right)/(N\times Kroot)]\times 100$$

in which *n_i_* = number of stems or roots in the DS development stage *i*, *st_i_* = value of the DS stage (1–4 and 0–4 for stems and roots, respectively), *N* = total number of plants assessed, and *K* = largest scale level (4 in all cases) [58]. Furthermore, for each combination of soil and treatment, the incidence of disease (DI; % of affected plants) and mortality (% of dead plants) were estimated at 28 days after inoculation.

Ungerminated seeds or plants with lesions on the hypocotyl, roots and/or stem were subjected to wet chamber and microscope preparations to confirm the identity of the inoculated pathogens.

### 4.8. Data Analyses

Data from the repetitions of each experiment were combined after checking for homogeneity of the experimental error variances by the *F* test (*p* ≥ 0.05). Subsequently, data were tested for normality and homogeneity of variances prior to conduct analyses of variance (ANOVA). For the dual culture assay, factorial ANOVA was conducted with MGI as dependent variable, and actinobacterial strains, fungal pathogens and their interaction as independent variables. Significant differences were observed for the two independent variables as well as for their interaction (*p* < 0.0001 in any cases). Thus, independent ANOVA were conducted to determine differences between actinobacterial strains against each fungal pathogen. For each fungal pathogen, mean values were compared using Tukey’s honestly significant difference (HSD) tests at *p* = 0.05 [61]. For the enzymatic activity, data of the halo (mm) for each of the three parameters evaluated were analyzed separately by the non-parametric Kruskal-Wallys test due to the assumptions of normality and homogeneity of variances were not fulfilled even though logarithmically, arcsine or square root transformation of the data were conducted. Data from the actinobacterial strains that not develop halo (0.0 mm) were excluded from the statistical analysis in any cases. Mean values were compared using Dunn’s comparisons test at *p* = 0.05. In the *in planta* experiment, data of total DS (seedlings inoculated with *M. phaseolina*), and DSstem and DSroot (seedlings inoculated with *R. solani*) were tested for normality and homogeneity of variances prior to conduct analyses of variance (ANOVA). Data from negative control were omitted since no symptoms were observed in all cases. For each dependent variable, a split-plot ANOVA was conducted with soil (*n* = 2) as main-plot factor and treatments (*n* = 6) as the subplot factor. Due to significant differences were observed in all cases for the two independent variables as well as for their interaction (*p* < 0.005), independent ANOVA were conducted to determine differences between treatments for each disease. The treatment means of total DS, or DSstem and DSroot were compared according to Fisher’s protected LSD test at *p* = 0.05 [61]. For both inoculated plants with *M. phaseolina* and *R. solani*, data on the final DI (% of affected plants) and mortality (% of dead plants) were analyzed by multiple comparisons for proportions tests at *p* = 0.05 [62]. Additionally, for plants inoculated with *R. solani*, the Pearson correlation coefficients (*r*) between the DSstem and DSroot were calculated using the average values of the two variables for each of the treatment evaluated in sterilized or non-sterilized soil (*n* = 6 in each type of soil). All data analyses were conducted using Statistix 10 [63].

## 5. Conclusions

The qualitative characterization of the extracellular enzyme activities, the antagonism of the *Streptomyces* spp. strains, as well as the in vivo studies against *M. phaseolina* and *R. solani* under semi-controlled conditions have allowed us to characterize promising strains as BCAs, and to have a biological alternative in the framework of the integrated management of the main common bean diseases caused by soil pathogens in Cuba. To confirm our laboratory results, the research should and will be evaluated under natural field conditions in further studies.

## Figures and Tables

**Table 1:** Antagonistic effect of the 60 actinobacterial strains on mycelial growth of *Macrophomina phaseolina* and *Rhizoctonia solani* in dual cultures.

Actinobacterial Strain	(MGI; %) ^a,b^	
	*Macrophomina phaseolina*	*Rhizoctonia solani*
CBQ-CB-14	60.9 ± 1.63	69.8 ± 1.40
CBQ-EA-2	70.4 ± 1.23	77.4 ± 1.20
CBQ-EA-12	46.2 ± 1.32	78.3 ± 0.37
CBQ-OSS-3	62.8 ± 1.55	61.2 ± 0.53
CBQ-ESFe-4	52.0 ± 2.00	63.7 ± 1.19
CBQ-CD-24	64.6 ± 1.48	75.4 ± 1.22
CBQ-EBa-5	60.5 ± 1.65	45.9 ± 1.32
CBQ-EBa-21	54.0 ± 1.11	55.7 ± 2.01
CBQ-Plat-2	66.6 ± 0.78	37.3 ± 1.91
CBQ-WP-14	56.7 ± 1.81	37.1 ± 0.40
CBQ-B-8	63.1 ± 1.54	69.0 ± 1.63
CBQ-J-4	28.1 ± 1.71	35.4 ± 2.70
CBQ-CB-3	40.5 ± 0.91	40.1 ± 1.35
CBQ-EA-3	44.4 ± 0.65	0.0 ± 0.00
CBQ-EB-27	0.0 ± 0.00	0.0 ± 0.00
CBQ-EC-3	32.2 ± 0.60	0.0 ± 0.00
CBQ-EC-18	36.0 ± 1.30	54.6 ± 0.91
CBQ-ECa-24	29.9 ± 1.51	0.0 ± 0.00
CBQ-ESFe-5	31.6 ± 1.31	0.0 ± 0.00
CBQ-ESFe-10	35.8 ± 1.25	0.0 ± 0.00
CBQ-ESFe-11	3.24 ± 1.01	0.0 ± 0.00
CBQ-ESFe-12	38.9 ± 1.32	43.5 ± 1.92
CBQ-Mg-6	8.7 ± 0.42	0.0 ± 0.00
CBQ-Ni-24	21.2 ± 0.58	0.0 ± 0.00
CBQ-Ni-32	5.9 ± 0.34	0.0 ± 0.00
CBQ-OSS-4	8.9 ± 0.30	14.3 ± 2.38
CBQ-Plat-3	20.4 ± 1.52	0.0 ± 0.00
CBQ-Plat-4	24.2 ± 1.43	0.0 ± 0.00
CBQ-SFe-5	4.0 ± 0.11	0.0 ± 0.00
CBQ-Wni-21	28.3 ± 1.55	0.0 ± 0.00
CBQ-RS-3	4.7 ± 0.19	0.0 ± 0.00
CBQ-A-2	0.0 ± 0.00	0.0 ± 0.00
CBQ-A-9	0.0 ± 0.00	52.7 ± 2.98
CBQ-A-17	9.7 ± 0.33	32.6 ± 2.30
CBQ-Amb-3	0.0 ± 0.00	0.0 ± 0.00
CBQ-B-1	0.0 ± 0.00	35.0 ± 1.73
CBQ-B-41	0.0 ± 0.00	21.5 ± 0.63
CBQ-B-44	0.0 ± 0.00	41.5 ± 0.43
CBQ-Be-29	0.0 ± 0.00	0.0 ± 0.00
CBQ-Be-36	0.0 ± 0.00	0.0 ± 0.00
CBQ-C-5	0.0 ± 0.00	5.6 ± 0.47
CBQ-C-7	0.0 ± 0.00	17.8 ± 1.04
CBQ-CB-6	17.8 ± 1.78	0.0 ± 0.00
CBQ-CD-12	0.0 ± 0.00	0.0 ± 0.00
CBQ-CD-19	3.4 ± 0.32	0.0 ± 0.00
CBQ-CD-21	38.2 ± 1.26	0.0 ± 0.00
CBQ-CD-23	26.6 ± 1.56	0.0 ± 0.00
CBQ-CD-25	3.5 ± 0.36	0.0 ± 0.00
CBQ-Cy-5	0.0 ± 0.00	0.0 ± 0.00
CBQ-CYM-2	0.0 ± 0.00	0.0 ± 0.00
CBQ-E-5	0.0 ± 0.00	0.0 ± 0.00
CBQ-EA-29	17.7 ± 0.31	0.0 ± 0.00
CBQ-EBa-1	0.0 ± 0.00	0.0 ± 0.00
CBQ-EB-5	0.0 ± 0.00	0.0 ± 0.00
CBQ-EBa-22	0.0 ± 0.00	44.3 ± 1.40
CBQ-EBe-3	15.0 ± 0.12	0.0 ± 0.00
CBQ-EBe-15	0.0 ± 0.00	0.0 ± 0.00
CBQ-EBe-16	0.0 ± 0.00	0.0 ± 0.00
CBQ-EBe-19	16.1 ± 0.49	0.0 ± 0.00
CBQ-EBe-20	0.0 ± 0.00	0.0 ± 0.00
CBQ-EC-5	20.0 ± 0.78	53.2 ± 0.98
HSD_0.05_	6.7	8.5

^a^ Mycelial Growth Inhibition (MGI; %) for *Macrophomina phaseolina* isolate CCIBP-Mp1 and *Rhizoctonia solani* isolate CCIBP-Rh1 were obtained by dual culture assays on PDA at 30 °C for 7 days in darkness. Data represent the average of twelve Petri dishes for each BCA or control ± the standard error of the means. ^b^ For each pathogen, significant differences between treatment means of MGI are given by a critical value for means comparison [HSD_0.05_ = 6.7 and 8.5% for *M. phaseolina* and *R. solani*, respectively] according to Tukey’s honestly significant difference (HSD) tests at *p* = 0.05.

**Table 2:** Chitinolytic, cellulolytic and proteolytic activity of the 31 actinobacterial strains selected for these experiments.

Actinobacterial Strain	Chitinolytic Halo (mm) ^a^	Cellulolytic Halo (mm) ^b^	Proteolytic Halo (mm) ^c^
CBQ-B-8	32.8 ± 0.96 ab	90.0 ± 0.41 a	41.3 ± 0.48 abcd
CBQ-CB-3	0.0 ± 0.0 c	49.3 ± 0.48 ab	0.0 ± 0.0 e
CBQ-CB-14	32.5 ± 2.65 ab	90.0 ± 0.41 a	50.8 ± 2.53 abc
CBQ-CD-24	25.3 ± 0.96 b	63.0 ± 2.38 ab	34.5 ± 0.50 abcd
CBQ-EA-2	34.0 ± 1.41 a	86.3 ± 0.48 ab	44.8 ± 1.65 abcd
CBQ-EA-3	31.3 ± 0.96 ab	36.3 ± 0.75 b	31.8 ± 0.75 bcd
CBQ-CB-4	0.0 ± 0.0 c	86.3 ± 0.48 ab	0.0 ± 0.0 e
CBQ-EA-12	31.3 ± 3.20 ab	80.0 ± 0.71 ab	51.5 ± 1.50 a
CBQ-EBa-5	33.5 ± 1.91 a	85.3 ± 1.18 ab	42.3 ± 0.25 abcd
CBQ-EB-27	0.0 ± 0.0 c	0.0 ± 0.0 c	0.0 ± 0.0 e
CBQ-EBa-21	29.8 ± 1.26 ab	68.5 ± 1.19 ab	47.8 ± 1.4 abc
CBQ-EC-3	0.0 ± 0.0 c	40.0 ± 0.71 ab	0.0 ± 0.0 e
CBQ-EC-18	29.3 ± 0.96 ab	0.0 ± 0.0 c	0.0 ± 0.0 e
CBQ-ECa-24	0.0 ± 0.0 c	90.0 ± 0.41 a	27.0 ± 0.71 d
CBQ-ESFe-4	24.8 ± 0.50 b	82.3 ± 2.59 ab	0.0 ± 0.0 e
CBQ-ESFe-5	0.0 ± 0.0 c	88.8 ± 0.75 ab	41.3 ± 2.39 abcd
CBQ-ESFe-10	31.3 ± 0.96 ab	81.8 ± 1.80 ab	0.0 ± 0.0 e
CBQ-ESFe-11	32.3 ±2.90 ab	84.5 ± 1.55 ab	0.0 ± 0.0 e
CBQ-ESFe-12	29.0 ± 1.41 ab	90.0 ± 0.41 a	49.0 ± 0.71 ab
CBQ-J-4	27.0 ± 1.41 ab	45.0 ± 1.41 ab	27.3 ± 0.48 d
CBQ-Mg-6	0.0 ± 0.0 c	62.0 ± 0.71 ab	44.8 ± 0.48 abcd
CBQ-Ni-24	27.3 ± 0.96 ab	88.8 ± 0.75 ab	31.8 ± 025 cd
CBQ-Ni-32	29.8 ± 0.50 ab	90.0 ± 0.41 a	37.3 ± 0.48 abcd
CBQ-OSS-3	33.0 ± 4.8 ab	67.0 ± 0.71 ab	39.3 ± 0.48 abcd
CBQ-OSS-4	0.0 ± 0.0 c	0.0 ± 0.0 c	44.0 ± 0.71 abcd
CBQ-Plat-2	29.8 ± 0.50 ab	90.0 ± 0.41 a	44.3 ± 0.75 abcd
CBQ-Plat-3	0.0 ± 0.0 c	90.0 ± 0.41 a	32.8 ± 0.25 abcd
CBQ-Plat-4	31.3 ± 0.96 ab	90.0 ± 0.41 a	33.5 ± 0.50 abcd
CBQ-SFe-5	28.3 ± 3.36 ab	47.3 ± 0.75 ab	0.0 ± 0.0 e
CBQ-Wni-21	0.0 ± 0.0 c	42.3 ± 0.48 ab	36.5 ± 0.95 abcd
CBQ-WP-14	29.8 ± 0.50 ab	90.0 ± 0.41 a	44.0 ± 0.71 abc

^a,b,c^ Halo develop (mm) for each actinobacterial strain grown onto chitin agar medium Colloidal, Yeast Extract-Malt Extract Agar (ISP2) plates with cellulose (1%, *w*/*v*), and ISP2 agar with 1% skimmed milk, respectively, at 28–30 °C in darkness for seven days. For each strain, data represent the average of twelve Petri dishes ± the standard error of the means. In each column, means followed by a common letter do not differ significantly according to Dunn’s multiple comparisons for proportions test at *p* = 0.05.

**Table 3:** Macroscopic characteristics of colonies of 11 actinobacterial strains (*Streptomyces* spp.) grown on Casein Starch Agar at 28 °C in darkness for 10 days *.

Actinobacterial Strain Code	Gram Stain ^a^	Aerial Mycelium ^b^	Color	Shape	Elevation	Edge	Consistency	Pigment
CBQ-B-8	+	+	White	Circular	Convex	Full	Hard	Yellow
CBQ-CB-14	+	+	White	Circular	Convex	Full	Hard	Yellow
CBQ-CD-24	+	+	White	Irregular	Convex	Whole	Hard	Beige
CBQ-EA-2	+	+	White	Circular	Convex	Lobed	Hard	Yellow
CBQ-EA-12	+	+	White	Circular	Pulvini	Whole	Hard	Brown
CBQ-EBa-5	+	+	Yellow	Irregular	Convex	Lobular	Hard	Yellow
CBQ-EBa-21	+	+	White	Irregular	Pulvini	Whole	Hard	Orange
CBQ-ESFe-4	+	+	Yellow	Circular	Convex	Lobed	Hard	Beige
CBQ-J-4	+	+	White	Circular	Convex	Whole	Hard	Beige
CBQ-OSS-3	+	+	Yellow	Irregular	Convex	Lobular	Hard	Yellow
CBQ-Plat-2	+	+	White	Circular	Convex	Whole	Hard	Yellow

^a^ (+):actinobacteria G+. ^b^ (+): Presence of aerial mycelium. * The phenotypic characteristics of the colonies of the actinobacterial strains were selected according with [18,19].

**Table 4:** Biochemical test results of the 11 actinobacterial strains selected for this experiment.

Biochemical Parameters *	Actinobacterial Strain Code										
	CBQ-B-8	CBQ-CB-14	CBQ-CD-24	CBQ-EA-2	CBQ-EA-12	CBQ-EBa-5	CBQ-EBa-21	CBQ-ESFe-4	CBQ-J-4	CBQ-OSS-3	CBQ-Plat-2
Catalase production	+	+	+	+	+	+	+	+	+	+	+
Lactose Fermentation	+	+	+	+	-	-	-	+	-	+	-
Glucose Fermentation	+	+	+	+	+	D	-	-	+	D	+
Mannitol Fermentation	+	+	-	+	-	+	-	+	-	-	-
Dextrose Fermentation	-	-	+	+	D	-	D	+	-	-	+
Fructose Fermentation	+	+	+	+	+	+	+	+	-	-	+
Maltose Fermentation	+	+	+	+	+	+	+	D	+	+	-
Sucrose Fermentation	+	+	+	+	+	+	+	D	+	-	-
Xylose Fermentation	+	+	-	+	+	-	+	+	-	+	+
Raffinose Fermentation	+	+	+	D	-	-	+	+	+	+	D
Casein Hydrolysis	-	-	-	+	-	+	-	-	+	+	-
Citrate Utilization	+	+	+	+	+	+	+	+	+	+	+
Urea Hydrolysis	+	+	+	+	+	+	+	+	+	+	+
Nitrate reduction	+	+	+	+	+	+	+	+	+	+	+
Indole production	-	-	-	-	-	-	-	-	-	-	-
Methyl red	+	+	-	+	-	+	+	-	-	-	+
Voges Proskauer	-	-	-	-	-	-	-	-	-	-	-
Gelatin hydrolysis	+	+	+	+	-	+	+	-	+	+	+
Starch hydrolysis	+	+	+	+	+	+	+	+	+	-	-

* (+): Positive reaction; (-): Negative reaction; (D): Dubious.

**Table 5:** Identification by sequencing the 16S rDNA gene of the two actinobacterial strains selected for molecular characterization with their corresponding GenBank accession numbers and data of Blast results obtained from GenBank.

Species	Isolate	Genbank Accession ^a^	Blast Accession ^b^	Query Length	Gaps ^c^	Identities ^d^	Maximum Identity (%)
*Streptomyces* sp.	CBQ-EA-2	OM417233	MT540570	1437	3/1390	1386/1390	99.71
*Streptomices* sp.	CBQ-B-8	OM417234	EU263063	1491	1/1491	1490/1491	99.93

^a^ Corresponding GenBank accession numbers of our isolates. ^b^ GenBank accession numbers blasted with the isolates obtained in this study. ^c^ Number of spaces introduced into the alignment to compensate for insertions and deletions in our sequence relative to blasted sequences. ^d^ Number of nucleotides of our sequences/Number of nucleotides of blasted sequences.

**Table 6:** Origen of actinobacterial strains used in this study.

Strain *	Isolation Substrate	Origin (Location, State)	Year of Collection
CBQ-RS-3	Sediment	River Seibabo, Villa Clara	2007
CBQ-A-2	Rhizosphere	Arco Iris, V. Clara	2007
CBQ-EA-2 ^a–c^	Endophytic, stem of *Mosiera bullata*	Arco Iris, V. Clara	2008
CBQ-B-8 ^a–c^	Rhizosphere, Carbonated brown	Botanical Garden UCLV, V. Clara	2008
CBQ-J-4 ^b,c^	Rhizosphere Ferrallitic red	River Seco, Jibacoa, Manicarargua. V. Clara	2008
CBQ-A-9	Rhizosphere	Arco Iris, V. Clara	2008
CBQ-A-17	Rhizosphere	Arco Iris, V. Clara	2008
CBQ-C-5	Rhizosphere	Cienfuegos	2008
CBQ-C-7	Rhizosphere	Cienfuegos	2008
CBQ-B-1	Rhizosphere	Botanical GardenUCLV, V. Clara	2008
CBQ-E-5	‘Fangos de Elguea’	Corralillo, V. Clara	2009
CBQ-B-41	Rhizosphere	Botanical Garden UCLV, V. Clara	2009
CBQ-B-44	Rhizosphere	Botanical Garden UCLV, V. Clara	2009
CBQ-Be-29	Rhizosphere	Escambray, Bernal	2010
CBQ-EC-3 ^b^	Endophytic	Coge Finca, Camajuaní, V. Clara	2010
CBQ-EC-5	Endophytic, stem of *Petiveria alliacea*.	V. Clara	2010
CBQ-Be-36	Rhizosphere	Escambray, Bernal	2010
CBQ-EBe-3	Endophytic, root of *Hibiscus elatus*	Bernal, Herradura, Manicarargua, V. Clara	2010
CBQ-Cy-5	Rhizosphere	Key I, V. Clara	2010
CBQ-CYM-2	Rhizosphere	Salinas, V. Clara	2010
CBQ-EA-29	Endophytic, stem	Arco Iris, V. Clara	2011
CBQ-EBa-1	Endophytic, root	Banao, S. Spíritus	2011
CBQ-EB-5	Endophytic	Botanical Garden UCLV, V. Clara	2011
CBQ-EBa-22	Endophytic, stem	Banao, S. Spíritus	2011
CBQ-EBe-15	Endophytic, root	Planta Escambray, Bernal	2011
CBQ-EBe-16	Endophytic, root	Planta Escambray, Bernal	2011
CBQ-EBe-20	Endophytic, root	Planta Escambray, Bernal	2011
CBQ-EA-3 ^b^	Endophytic	Arco Iris. V. Clara	2011
CBQ-EB-27 ^b^	Endophytic, Stem	Jandín Botánico UCLV, V. Clara	2011
CBQ-EA-12 ^a,b^	Endophytic, leaf of *Mosiera bullata*,	Arco Iris. V. Clara	2011
CBQ-EC-18 ^b^	Endophytic, stem of *Petiveria alliacea*.	Coge Finca, Camajuaní, V. Clara	2011
CBQ-ECa-24 ^b^	Endophytic, root	Caguanes, S. Spíritus	2011
CBQ-EBa-5 ^a,b^	Endophytic, root	Banao. S. Spíritus	2011
CBQ-EBa-21 ^a,b^	Endophytic, root of *Piper aducum*	Banao. S. Spíritus	2011
CBQ-Ni-24 ^b^	Endophytic, stem	Nicho, V. Clara	2011
CBQ-Ni-32 ^b^	Endophytic, stem	Nicho, V. Clara	2011
CBQ-ESFe-12 ^b^	Endophytic, stem of *Fleurya cuneata,*	Loma Sta Fé, V. Clara	2012
CBQ-ESFe-5 ^b^	Endophytic, stem of *Fleurya cuneata,*	Loma Sta Fé, V Clara	2012
CBQ-ESFe-10 ^b^	Endophytic, stem of *Fleurya cuneata,*	Loma Sta Fé, V. Clara	2012
CBQ-ESFe-11 ^b^	Endophytic, stem of *Fleurya cuneata,*	Loma Sta Fé, V. Clara	2012
CBQ-WP-14 ^b^	Sediment, *Clarias batrachus*,	V. Clara	2012
CBQ-ESFe-4 ^a,b^	Endophytic, leaf of *Piper aduncum*	Loma Santa Fé. V. Clara	2012
CBQ-Wni-21 ^b^	Sediment	River Nicho, V. Clara	2012
CBQ-Amb-3	Endophytic, Stem of *Cecropia adenopu*,	V. Clara	2013
CBQ-CB-14 ^a,b^	Sediment	Caves de Bellamar, Matanzas	2013
CBQ-OSS-4 ^b^	Endophytic	Topes de Collantes, S. Spíritus	2013
CBQ-Plat-3 ^b^	Endophytic, Stem of *Comocladia platyphylla.*	V. Clara	2013
CBQ-Plat-4 ^b^	Endophytic, Root of *Comocladia platyphylla,*	V. Clara	2013
CBQ-CB-3 ^b^	Sediment	Caves de Bellamar, Matanzas	2013
CBQ-Plat-2 ^a,b^	Endophytic, stem of *Comocladia platyphylla.*	V. Clara	2013
CBQ-OSS-3 ^a,b^	Endophytic, leaf of *Ossanum*	Topes de Collantes, S. Spíritus	2013
CBQ-CB-4 ^b^	Sediment	Caves de Bellamar, Matanzas	2013
CBQ-CD-12	Rhizosphere	Key Las Dunas, V. Clara	2014
CBQ-CD-19	Rhizosphere	Key Las Dunas, V. Clara	2014
CBQ-CD-21	Rhizosphere	Key Las Dunas, V. Clara	2014
CBQ-CD-23	Rhizosphere	Key Las Dunas, V. Clara	2014
CBQ-CD-25	Rhizosphere	Key Las Dunas, V. Clara	2014
CBQ-CD-24 ^a,b^	Rhizosphere	Key Las Dunas. V. Clara	2014
CBQ-Mg-6 ^b^	Endophytic, stem of *Rhizophora mangle*	Mégano, La Habana	2014
CBQ-SFe-5 ^b^	Rhizosphere	Sta Fé, V. Clara	2015

^a^ Strains selected for biochemical characterization. ^b^ Strains selected for qualitative determination of their chitinolytic, cellulolytic and proteolytic activity. ^c^ Strains selected for *in planta* bioassays. * All actinobacterial strains used in this study were collected in Cuba by Dr. C. R. Medina-Marrero (CBQ: ‘Centro de Bioactivos Químicos’).

## Data Availability

The data that support the findings of this study are available from the corresponding author upon reasonable request.
